# Supplementary figures and images for: Experimental Evolution Reveals Redox State Modulates Mycobacterial Pathogenicity
Source: Front Genet. 2022 Mar 16;13:758304. doi: 10.3389/fgene.2022.758304 (PMC8965865; doi:10.3389/fgene.2022.758304)

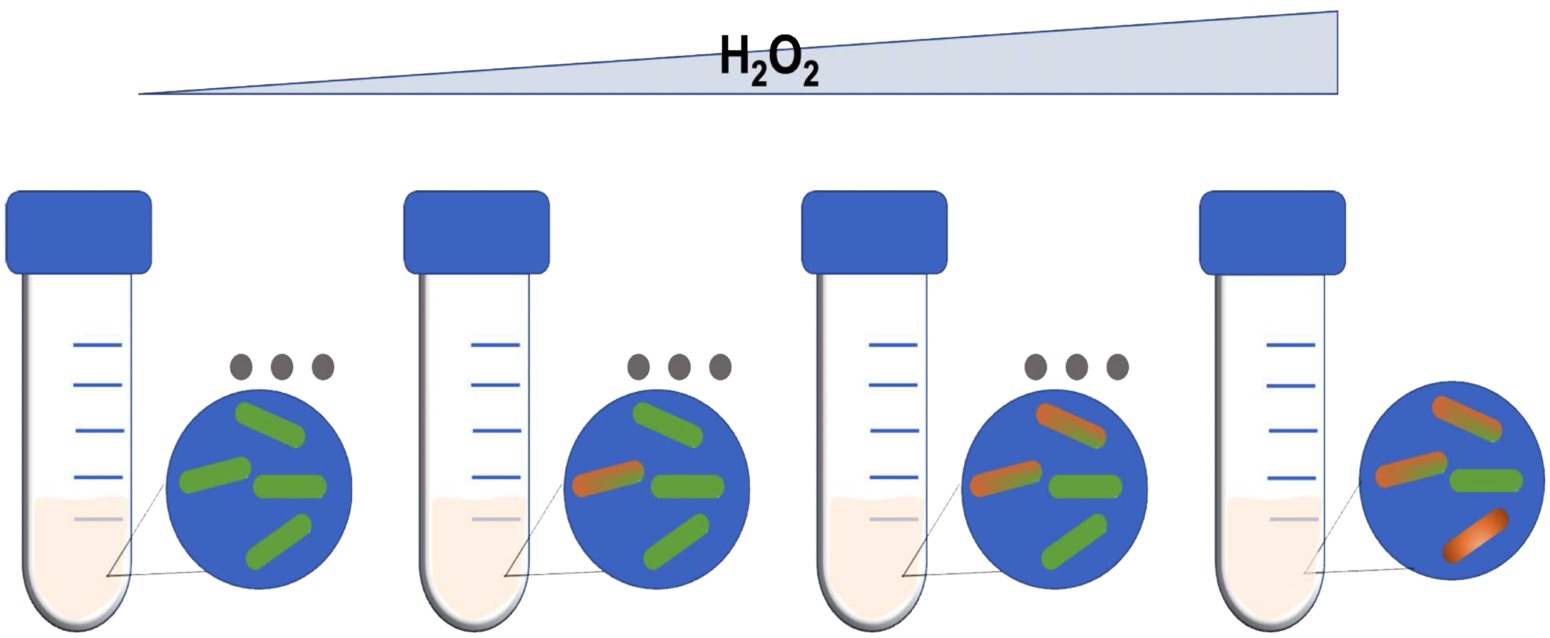

Supplement: Supplementary file 1 [file Image1.jpeg]
